# On the Association between Implant-Supported Prosthesis and Glycemic Control (HbA1c Values)

**DOI:** 10.3390/ijerph19116923

**Published:** 2022-06-06

**Authors:** Daya Masri, Hiba Masri-Iraqi, Joseph Nissan, Carlos Nemcovsky, Leon Gillman, Sarit Naishlos, Liat Chaushu

**Affiliations:** 1 Department of Oral and Maxillofacial Surgery, Rabin Medical Center, Petach-Tikva 4941492, Israel; dr.dayamasri@gmail.com (D.M.); gillman.leon@gmail.com (L.G.); 2 Department of Endocrinology, Rabin Medical Center, Petach-Tikva 4941492, Israel; iraqihiba@yahoo.com; 3 Department of Oral-Rehabilitation, Rabin Medical Center, Petach-Tikva 4941492, Israel; nissandr@gmail.com; 4 The Maurice and Gabriela Goldschleger School of Dental Medicine, Tel Aviv University, Tel Aviv 6997801, Israel; 5 Department of Periodontology and Oral Implantology, The Maurice and Gabriela Goldschleger School of Dental Medicine, Tel Aviv University, Tel Aviv 6997801, Israel; carlos@tauex.tau.ac.il; 6 Department of Pedodontics, The Maurice and Gabriela Goldschleger School of Dental Medicine, Tel Aviv University, Tel Aviv 6997801, Israel; river554@gmail.com

**Keywords:** HbA1c, implant supported prosthesis (ISP), glycemic control, edentulism, dental implants, early implant failure (EIF)

## Abstract

*Background*: Dietary habits, food intake and oral health are important factors for general health. The aim of these present study was to assess the association between implant-supported fixed oral rehabilitation and glycemia, by monitoring HbA1c values before and after implant-supported prostheses (ISP) delivery to diabetic individuals. *Methods:* Retrospective, cohort study based on dental records. All treatments were performed by experienced oral and maxillofacial surgeons and experienced prosthodontists. Inclusion criteria: ISP delivery, diagnosis of diabetes in the medical files, consecutive individuals. Variables included—primary outcome—differences (delta) in HbA1c values prior to implant placement and one year after ISP delivery, early implant failure (EIF). Confounding factors included age, gender, physical status, smoking, implant jaw location, implant length, implant width, total implant count per individual. *Results:* Statistically significant (*p* < 0.01) decrease in HbA1c from 7.10 ± 1.09% to 6.66 ± 1.02% following ISP delivery was recorded. The mean HbA1c delta was 0.44 ± 0.73%, where 39.0% of the patients had a significant improvement (delta decrease > 0.5%). Univariate and multivariate model using logistic regression at individual level showed that initial high HbA1c levels was the only factor positively predicting improvement (OR = 1.96, CI [1.22, 3.14], *p* < 0.01). Univariate model at implant level demonstrated that implants placed in the anterior maxilla also contributed to significant improvement in HbA1c values. Multivariate analysis at implant level was similar to individual level. Number of missing teeth did not affect the results significantly. *Conclusion:* ISP delivery to partially or completely edentulous diabetic individuals may improve HbA1c balance. The mechanism awaits future elucidation.

## 1. Introduction

Diabetes is estimated to affect approximately 10% (95% with type 2 diabetes) of the population [1,2]. It is defined as elevated levels of glycemia (glucose and glycated hemoglobin) [1,2]. Hemoglobin A1c, (HbA1c) is the most widely used clinical test to estimate mean blood glucose. It is used for diagnosis and monitor the treatment efficacy. HbA1c is often used in clinical trials demonstrating the benefits of different diabetic drugs on improved glycemic control on and its effect on microvascular and macrovascular outcomes. Based on US Food and Drug Administration (FDA) requirements, it is the primary endpoint for the demonstration of glycemia-lowering efficacy for new diabetes drugs [2].

Chewing is the action by which the nutrients are processed in the oral cavity using muscles, soft tissues, jaws and teeth [3]. Proper and satisfying food cutting and chewing depends on the number of teeth in the dentition. Individuals with missing dentition chew worse. They use larger number of chewing cycles and swallow bigger food particles [4].

“Masticatory function” may be either objective (“masticatory performance”) or subjective capacity (“masticatory ability”). Objective masticatory function can be assessed by the individual’s capacity to grind test food after a fixed number of chewing cycles. Masticatory performance is reduced in people who have lost premolar/molar teeth and in those who wear removable dentures. Implant-supported prostheses improve the masticatory function both objectively and subjectively in edentulous patients [3,4].

Maximum voluntary “bite force” is an additional important factor. Bite force has a large influence on masticatory performance in subjects with overdentures, full dentures as well as natural dentitions. Bite force explains over 60% of the variance in masticatory performance. Maximum bite force is influenced by factors like jaw location (incisors vs. premolars), number of teeth included, and unilateral versus bilateral measurements. Significantly higher values are reported in men vs. women and decrease with age. The decrease in bite force may be due to direct effect on muscle strength, or changes in food choice because of deteriorated dentition. This may lead to less-trained jaw muscles [4].

In partially edentulous patients numbers of missing teeth and chewing ability may have a metabolic systemic effect expressed in basal metabolic index (BMI), high-sensitivity C-reactive Protein (hs-CRP) test, and HbA1c values [5].

Edentulous patients have a lower masticatory power and efficacy compared with dentate patients, which makes them select soft and liquid food that is easy to chew [6]. Such selection may negatively impact glycemic control, first and foremost in diabetic patients.

Teeth loss is also a significant contributing factor for obesity [7]. Both obesity and HbA1c values affect the diagnosis and prognosis of diabetes [8].

Dietary habits, food intake and oral health are important factors for general health, but the reciprocal effect of rehabilitating oral function and relationship with overall nutritional status are still under research [2,9].

Implant-supported prosthesis (ISP) is usually employed in partial or full edentulism. However, peri-implant disease may occur. Peri-implant health is absence of signs of inflammation as bleeding on probing [10,11,12,13,14,15,16]. Peri-implant mucositis was defined by bleeding on probing and visual signs of inflammation. Peri-implantitis was defined as a plaque-associated pathologic condition occurring in the tissue around dental implants, characterized by inflammation in the peri-implant mucosa and subsequent progressive loss of supporting bone. Peri-implantitis, in the absence of treatment, seems to progress in a non-linear and accelerating pattern. Surgical entry at peri-implantitis sites often reveals a circumferential pattern of bone loss. Data identifying diabetes as potential risk factors/indicators for peri-implantitis are inconclusive [14].

Improving general systemic health by restoring oral function is one of the main goals of oral rehabilitation of partially or completely edentulous patients. The purpose of the present study was to assess the impact of implant-supported oral rehabilitation on glycemia, by monitoring HbA1c values before and after implant-supported prostheses (ISP) delivery. The null hypothesis was that there is no difference in HbA1c values before and after ISP delivery.

## 2. Materials and Methods

The present retrospective, cohort study is based on dental records of the Department of Oral and Maxillofacial Surgery, Rabin Medical Center, Campus Beilinson, Israel. All treatments were performed by experienced oral and maxillofacial surgeons and experienced prosthodontics. Initial examination included medical history and clinical examination. Imaging used included periapical and panoramic radiographs and cone beam computerized tomography (CBCT). Treatment alternatives were thoroughly explained to the patients. All patients signed an informed consent. In partially edentulous patients an oral hygiene regimen was advocated prior to surgery. When bone grafting was required, a staged approach was used, followed by a 4–6 months’ waiting time prior to implant placement. Patients were treated either under local anesthesia in the department clinic or under general anesthesia in the operating room. One gram Amoxicillin was given 45 min prior to surgery. Chlorohexidine 0.12% rinses were used for 2 min immediately before first incision. Local anesthesia was always used. Surgery commenced using midcrestal and vertical releasing incisions. Following osteotomies, as instructed by implant manufacturer, implants were inserted. Initial stability was verified. Rough-surface (sand-blasted and acid-etched) implants were used. Soft tissue closure was performed using tension-free resorbable sutures. Postoperative antibiotics included Amoxicillin 500 mg TID. Analgesics included Etodolac 400 mg TID. Chlorhexidine 0.12% rinses were advocated for the first 2 weeks TID. Patients were seen weekly in the first month and monthly for the next 3 months. After 4 months second stage surgery (implant exposure) was performed. After 6 weeks waiting time for soft tissue healing prosthetic rehabilitation started. During the first-year post rehabilitation patients were seen every 3 months. From the second year of loading, they were seen yearly for clinical and radiological follow-up.

Data were extracted and manually screened twice by 2 examiners (DM and LC). The study protocol was approved by the ethics committee of the Rabin Medical Center, Campus Beilinson, Israel (0674-19rmc). The present script complies with the STROBE guidelines [17].

### 2.1. Data Collection

Inclusion criteria:

1. ISP delivery
2. Diagnosis of diabetes in the medical files
3. Consecutive individuals treated between January 2013–December 2018
4. Available data

Exclusion criteria:

1. Lack of data
2. Modification in medications for diabetes

Patients with co-morbidities as—head and neck irradiation, immunodeficiencies, immunosuppressive treatments, bone modulating agents for malignancy, and other absolute contraindications for implant dentistry, did not receive ISP and were thus excluded. Sample size was not calculated in advance since all patients receiving ISP between January 2013–December 2018 were included.

Variables for individual level included—

Primary outcome parameters—

1. HbA1c values prior to implant placement and one year after ISP delivery
2. Early implant failure (up to 1 year after ISP delivery) (EIF)

Confounding parameters included—

1. Age
2. Gender
3. Physical status according to American Society of Anesthesiology (ASA) [18]
4. Smoking
5. Number of implants per individual
6. Implant length/diameter
7. Implant location

### 2.2. Statistical Analysis

The data was analyzed using SPSS software version 25.0 (SPSS Inc., Chicago, IL, USA; STATA 15.1, StataCorp LLC, College Station, TX, USA). Sample size calculation was conducted using G-Power software under the following assumptions: type 1 error of 5%, minimum desired power of 80%, and a moderate effect size of the association between HbA1c levels and ISP delivery (OR = 2.0). Under these assumptions, the minimum sample required is 88 patients. Descriptive statistics were preformed using means and standard deviations with the continuous variables, and frequencies and rates for the discrete variables. Significant improvement in HbA1c levels was considered for delta scores > 0.5% [19]. The study group was further divided into those with vs. without significant improvement. Wilcoxon Sum Rank test was conducted to assess the significance of the change in HbAa1c from before and after the procedure. For independent comparisons between the two groups Mann-Whitney and Chi-square tests were used. Mann-Whitney tests were conducted for the continuous variables, and Chi-square for the discrete variables. Logistic regression was used for conducting multivariate model to predict which parameters may affect improvement. Significance was considered for *p*-values lower than 0.05.

## 3. Results

### 3.1. Demographic and Clinical Characteristics at Individual Level

Data was gathered from 100 individuals (Table 1). Average age was 67.16 ± 11.50 years. The individual’s age was further divided to 3 groups—≤65 years (45.0%); 66–79 years (42.0%); and ≥80 years (13.0%). Most were females (60.0%). The average total implant number per individual was 4.29 ± 2.85 implants. Only 6% of the patients were smokers. The physical status of most of the patients was ASA 2 (56.0%) and the rest ASA 3 (44.0%).

Differences in mean HbA1c values before and after ISP delivery for the entire group were assessed using Wilcoxon Sum Rank test. The results show that there was a statistically significant (*p* < 0.01) decrease in HbA1c from **7.10 ± 1.09% to 6.66 ± 1.02%** following ISP delivery. The mean HbA1c delta was 0.44 ± 0.73%, where 39.0% of the patients had a significant improvement (delta decrease > 0.5%).

Early implant failure at individual level (at least one failure) was 12.0%.

### 3.2. Univariate Analysis (Individual Level)

The difference of demographic and clinical characteristics affecting significant improvement after ISP delivery were assessed using Mann-Whitney tests. The results (Table 2) show that patients who had significant improvement had statistically significant higher mean HbA1c values before **(7.57 ± 1.14)** vs. after ISP delivery **(6.80 ± 0.95, *p* < 0.01)**. No statistically significant differences were noted in either primary outcome parameters—EIF, or confounding factors—age, gender, physical status, smoking status and total implant count per individual.

### 3.3. Multivariate Analysis (Individual Level)

Multivariate model was conducted using logistic regression. The results (Table 3) show that initial high HbA1c levels positively predict improvement **(OR = 1.96, CI [1.22, 3.14], *p* < 0.01)**.

### 3.4. Demographic and Clinical Characteristics (Implant Level)

Data (Table 4) was gathered from 428 implants. The average age was (66.56 ± 10.84 years). The individual’s age was further divided to 3 groups—≤65 years (48.2%); 66–80 years (40.7%); and ≥80 years (11.0%). Most the implants were placed in females (64.6%).

The implants’ average length and width were 11.43 ± 1.59 mm and 3.85 ± 0.56 mm, respectively.

The differences in HbAa1c levels before and after ISP delivery were assessed using Wilcoxon Sum Rank test. The results show that there was a statistically significant decrease (*p* < 0.01) from 7.24 ± 1.23% to 6.75 ± 1.12%. The average difference between pre and post ISP delivery of HBbA1c values was (0.49 ± 0.78%), where 38.8% of the implants had a significant (Delta HbA1C > 0.5%) improvement.

Early implant failure value was low (2.8%). Most (96.7%), of the implants were placed in non-smokers.

About eighth of the implants were placed in the anterior maxilla (13.8%) or posterior maxilla (15.5%), and fifth were placed in the premolar maxilla (20%). About a fifth of the cases were in the anterior mandible (17.7%), eighth in the posterior mandible (16.6%) or premolar mandibular area (16.4%).

The physical status of the individuals where most of the implants were placed was ASA 2 (56.2%) and the rest ASA 3 (43.8%).

### 3.5. Univariate Analysis (Implant Level, n = 428)

The effect of demographic and clinical characteristics was assessed using Mann-Whitney tests and Chi-square tests (Table 5). The results demonstrate statistically significant (*p* < 0.01) mean higher HbA1c initial values in cases where a significant (>0.5%) improvement was noted **(7.84 ± 1.17 vs. 6.87 ± 1.11)**. There was a statistically significant *(p* < 0.01) higher incidence of anterior maxillary implants where there was a significant improvement **(19.9% vs. 9.9%)**.

### 3.6. Multivariate Analysis (Implant Level)

The results (Table 6) show that HbA1c level before the ISP delivery positively predict improvement (**OR = 1.82, CI [1.47, 2.25], *p* < 0.01**).

### 3.7. The Effect of Number of Missing Teeth

A total of 73/100 patients (Table 7) improved their HbA1c values, meaning improvement of their glycemic control, while 39/73 improved their HbA1c significantly (>0.5%). Eleven patients showed no change while 16 patients showed deterioration in their HbA1C value. Merely 5 individuals showed significant deterioration. There was no statistically significant difference between the groups.

## 4. Discussion

Diabetes is a systemic condition with a major effect on the course of periodontitis. Diabetes-associated periodontitis should not be regarded as a distinct diagnosis, but diabetes should be recognized as an important modifying factor and included in a clinical diagnosis of periodontitis as a descriptor [10,20].

Implant survival rate for dental rehabilitation in diabetics do not differ from healthy patients within the first 6 years, but in the long-term observation up to 20 years, a reduced implant survival can be found in diabetic patients. Osseointegration following implantation—after 1 year, there is no difference between diabetic and healthy individuals, not even to the poorly controlled HbA1c. There seems to be no elevated risk of peri-implantitis in the short but in the long-term, peri-implant inflammation seems to be increased in diabetic patients. To improve implant survival and reduce postoperative complications, supportive therapy consisting of prophylactic antibiotics and chlorhexidine mouth rinse is recommended [21].

The impact of fixed oral rehabilitation on the nutritional status in general and on glycemic control in particular is scarce. To the best of our knowledge, this is the first study to assess retrospectively the impact of ISP delivery on glycemic control by monitoring HbA1c values. The present study cohort is a significant sample of diabetic individuals (n = 100) and a large number of dental implants inserted (n = 428). EIF was 2.8% on implant level and nearly 12% on patient level. Looking at the entire cohort EIF are within the range reported in the literature [22,23].

The results suggest, in a statistically significant manner, that oral rehabilitation with ISP may improve glycemic control (HbA1c values).

Uni and multivariate analysis at individual level for predicting improvement showed that initial high HbA1C values may predict improvement. At implant level the results are similar. Univariate analysis showed that the initial high HbAa1c values and implant location (anterior maxilla) may predict improvement. Multivariate analysis showed initial high HbAa1c is the only predictive factor.

Shinkai et al. [24] and Gunji et al. [25] reported that no significant differences were found in the diet quality of subjects with dentures of good vs. poor technical quality. Replacement of old, poor-fitting dentures with new conventional complete dentures improved masticatory performance but not dietary intake. The present study demonstrated that dietary intake (HbA1c values) was improved by ISP delivery. The difference in the outcome may be related to the delivery of fixed prosthesis in the present study, in contrast to the two previous studies who reported removable prostheses or it may be speculated that masticatory ability and not only masticatory performance is improved leading to a better masticatory function.

Oral rehabilitation can contribute to glycemic control in two aspects. First, the transition from sugary liquid food to solid one (meat, fruit and vegetables) increases masticatory cycles and can activate the satiety system contributing to glycemic control. Second, a marked improvement in the masticatory function [4,26].

Accumulating studies indicate and ensure that increasing masticatory function influences post-prandial subjective appetite [27]. In a systematic review and meta-analysis, who assessed the influence of mastication on satiety and food intake, found that chewing may decrease self-reported hunger and food intake, possibly, through alterations in gut hormones responses related to satiety. A study of Cassady et al. [28] demonstrated that increasing the number of chewing cycles before swallowing almonds increased post-prandial G glucagon-like peptide-1 (GLP-1), one of the main gut peptides influencing satiety sense and insulin secretion. This class of medications is used for the treatment of type 2 diabetes. Studies by Li et al. [29] and Zhu and Hollis [30] suggest that increasing the number of chewing cycles, increased Cholecystokinin (CCK) which also acts as a hunger suppressant and reduced Ghrelin (often called a “hunger hormone” because it increases food intake).

It can be speculated that rehabilitation of missing teeth may help selecting food like fruits, vegetables and more protein containing food like meats such a change will increase mastication cycles compared with the choice of soft and liquid food (easy to chew). Such selection may positively impact glycemic control, first and foremost in diabetic patients by supplement the body with appropriate caloric intake which will enhance the number of mastication cycles and positively impact the gut hormones.

In the present study a substantial improvement in HbA1c values was demonstrated. However, it is impossible to explain from the data the exact mechanism. If masticatory performance is improved then we should have seen better results with less existing dentition. Unfortunately, the results were not statistically significant (Table 7). Masticatory ability may be another reason. With new ISP, the patients’ confidence may lead them to choose improved food quality. The data does not answer this question. Maximum bite force improvement is another option. The significance of anterior teeth in the univariate analysis supports improvement in bite force which may contribute up to 60% of masticatory performance. Age and gender did not affect bite force. Realistically it is most probably a multifactorial mechanism that remains to be elucidated in future studies.

Additional means may be taken to help for the professional and home management of diabetic patients, as mentioned in long studied by the research group of Scribante et al. as the use of ozonated water [31], postbiotics [32] and paraprobiotics [33].

The present study has several limitations. First, retrospective cohort study. More prospective studies have to be held. Second, changes in diet and food patterns should be monitored and demonstrated following oral rehabilitation. Third, the impact of partial edentulism length and location and complete edentulism should be prospectively evaluated. Forth, the effect of masticatory performance, masticatory ability and bite force requires specific evaluation.

## 5. Conclusions

ISP delivery in partially or completely edentulous diabetic individuals may contribute to glycemic control. Partially or completely edentulous patients are encouraged to rehabilitate their teeth with ISP in order to improve their glycemic control.

## Figures and Tables

**Table 1:** Demographic and clinical characteristics (individual level).

	M	SD	N	%
Age	67.16	11.50		
Age groups				
≤65			45	45.0
66–79			42	42.0
≥80			13	13.0
Gender				
Female			60	60.0
Male			40	40.0
Total implant number	4.29	2.85		
ASA				
ASA 2			56	56.0
ASA 3			44	44.0
HbA1c before	7.10	1.09		
HbA1c after	6.66	1.02		
Delta HbA1c	0.44	0.73		
HbA1c improvement			39	39.0
Implant failure			12	12.0
Smoking			6	6.0

**Table 2:** Univariate analysis (individual level).

	No Significant Improvement (N = 61)		Significant Improvement (N = 39)		Χ^2^	*p*
	M ± SD	N (%)	M ± SD	N (%)		
Age	68.10 ± 10.40		65.69 ± 13.04			0.51
Age groups					0.58	0.75
−65		26 (42.6)		19 (48.7)		
66–79		26 (42.6)		16 (41.0)		
+80		9 (14.8)		4 (10.3)		
Gender					0.34	0.56
Female		38 (62.3)		22 (56.4)		
Male		23 (37.7)		17 (43.6)		
Total implant count	4.29 ± 2.83		4.28 ± 2.92			0.93
ASA					0.00	0.95
ASA 2		34 (55.7)		22 (56.4)		
ASA 3		27 (44.3)		17 (43.6)		
**Ha1c before**	**6.80 ± 0.95**		**7.57 ± 1.14**			**<0.01**
Ha1c after	6.78 ± 1.05		6.47 ± 0.95			0.13
EIF		7 (11.5)		5 (12.8)	0.04	0.84
Smoking		4 (6.6)		2 (5.1)	0.09	0.99

**Table 3:** Multivariate analysis (individual level).

	B	OR	CI		*p*
			L	H	
Age	−0.02	0.98	0.94	1.02	0.37
Gender (Male)	0.33	1.39	0.52	3.74	0.51
Total implant count	−0.07	0.93	0.79	1.10	0.41
**HbA1c before**	**0.67**	**1.96**	**1.22**	**3.14**	**<0.01**
Implant failure	−0.49	0.61	0.13	2.51	0.53
Smoking	−0.20	0.82	0.12	5.56	0.83
ASA (3)	−0.09	0.91	0.32	2.62	0.87

**Table 4:** Demographic and clinical characteristics (implant level, n = 428).

	M	SD	N	%
Age	66.56	10.84		
Age groups				
≤65			206	48.1
66–79			175	40.9
≥80			47	11.0
Gender				
Female			277	64.7
Male			151	35.3
Implant Length	11.43	1.59		
Implant Width	3.85	0.56		
Ha1c before	7.24	1.23		
Ha1c after	6.75	1.12		
Delta HbA1c	0.49	0.78		
HbA1c improvement			166	38.8
EIF			12	2.8
Smoking			14	3.3
Anterior maxilla			59	13.8
Premolar maxilla			86	20
Posterior maxilla			66	15.5
Anterior mandible			76	17.7
Premolar mandible			70	16.4
Posterior mandible			71	16.6
ASA				
ASA 2			241	56.2
ASA 3			187	43.8

**Table 5:** Univariate analysis (implant level).

	No Significant Improvement (N = 262)		Significant Improvement (N = 166)		X^2^	*p*
	M ± SD	N (%)	M ± SD	N (%)		
Age	67.11 ± 9.93		65.69 ± 12.11			0.65
Age groups					1.84	0.40
≤65		125 (47.5)		82 (49.4		
66–79		104 (39.8)		70 (42.2)		
≥80		33 (12.6)		14 (8.4)		
Gender					0.07	0.79
Female		171 (65.1)		106 (63.9)		
Male		91 (34.9)		60 (36.1)		
Implant Length	11.42 ± 1.54		11.46 ± 1.66			0.94
Implant Width	3.86 ± 0.68		3.83 ± 0.30			0.22
**Ha1c before**	**6.87 ± 1.11**		**7.84 ± 1.17**			**<0.01**
Ha1c after	6.84 ± 1.22		6.61 ± 0.94			0.45
Failure		7 (2.7)		5 (3.0)	0.04	0.84
Smoking		11 (4.2)		3 (1.8)	1.85	0.17
**Anterior maxilla**		**26 (9.9)**		**33 (19.9)**	**8.29**	**<0.01**
Premolar maxilla		52 (19.9)		34 (20.4)	0.20	0.89
Posterior maxilla		46 (17.5)		20 (12.0)	2.41	0.12
Anterior mandible		53 (20.3)		23 (13.9)	2.89	0.09
Premolar mandible		41 (15.6)		29 (17.5)	0.23	0.63
Posterior mandible		44 (16.8)		27 (16.3)	0.03	0.87
ASA					0.29	0.59
ASA 2		145 (55.2)		96 (57.8)		
ASA 3		117 (44.8)		70 (42.2)		

**Table 6:** Multivariate analysis (implant level).

	B	OR	CI		*p*
			L	H	
Age	−0.00	1.00	0.98	1.02	0.74
Gender (Male)	0.39	1.48	0.89	2.27	0.17
Implant Length	−0.05	0.95	0.80	1.12	0.52
Implant Width	−0.31	0.73	0.49	1.10	0.11
**HbA1c before**	**0.60**	**1.83**	**1.47**	**2.27**	**<0.01**
ASA (3)	−0.22	0.80	0.47	1.39	0.43
EIF	0.15	1.16	0.28	4.82	0.85
Smoking	−0.51	0.60	0.14	2.53	0.45
Anterior maxilla	0.08	1.09	0.12	9.45	0.93
Premolar maxilla	−0.28	0.75	0.09	6.48	0.81
Posterior maxilla	−0.63	0.53	0.06	4.82	0.59
Anterior mandible	−0.96	0.38	0.04	3.34	0.40
Premolar mandible	−0.30	0.74	0.09	6.38	0.83
Posterior mandible	−0.41	0.66	0.07	5.87	0.72

**Table 7:** Delta HbA1c values distribution according to jaw missing teeth.

Delta HbA1c Values	Number of Individuals (100)	≤3 Missing Teeth N (%) (43)	≥4 Missing Teeth N (%) (38)	Complete Edentulism N (%) (19)
Significant increase (>0.5%)	5	4 (9.3%)	1 (2.6%)	0 (0%)
Increase (0.1–0.49%)	11	1 (2.3%)	7 (18.4%)	3 (15.8%)
No change	11	7 (16.3%)	3 (7.9%)	1 (5.3%)
Decrease (0.1–0.49%)	34	16 (37.2%)	12 (31.6%)	6 (31.6%)
Significant Decrease (>0.5%)	39	15 (34.9%)	15 (39.5%)	9 (47.3%)

## Data Availability

The data presented in this study are available on request from the corresponding author. The data are not publicly available due to ethics.
